# Deep Learning Model as a New Trend in Computer-aided Diagnosis of Tumor Pathology for Lung Cancer

**DOI:** 10.7150/jca.43268

**Published:** 2020-03-26

**Authors:** Lei Cong, Wanbing Feng, Zhigang Yao, Xiaoming Zhou, Wei Xiao

**Affiliations:** 1 Department of Oncology, Shandong Provincial Hospital affiliated to Shandong University, Jinan, China; 2 Cheeloo College of Medicine, Shandong University, Jinan, China; 3 Department of Pathology, Shandong Provincial Hospital affiliated to Shandong University, Jinan, China; 4 Department of Scientific Research, Shandong Provincial Hospital affiliated to Shandong University, Jinan, China

**Keywords:** deep learning, pathology, lung cancer, artificial intelligence

## Abstract

Lung cancer is one of the main causes of cancer-related death in the world. The identification and characteristics of malignant cells are essential for the diagnosis and treatment of primary or metastatic cancers. Deep learning is a new field of artificial intelligence, which can be used for computer aided diagnosis and scientific research of lung cancer pathology by analyzing and learning through establishment and simulation of human brain. In this review, we will introduce the application, progress and problems of deep learning in pathology of lung cancer and make prospects for its future development.

## Introduction

Lung cancer is one of the most common cancers and one of the major causes of cancer-related deaths worldwide. The incidence and mortality of lung cancer are increasing year by year. The 5-year survival rate of lung cancer is as low as 18%. The 5-year survival rate of patients with advanced metastatic lung cancer is only 5%. The 5-year overall survival rate of patients with stage I non-small cell lung cancer is 77.9%. Therefore, accurate pathological diagnosis, so can receive precise treatment and improve prognosis, becomes an important and key link 1.

Computer-aided diagnosis (CAD) is a computerized procedure for pathological diagnosis of malignant tumors belongs to the category of artificial intelligence (AI), which assists the detection of tumor lesions through medical image processing technology and other possible physiological and biochemical means combined with analysis and calculation. In the medical field, AI can transform qualitative subjective image information into quantitative objective image information, and help clinicians to supplement clinical decision-making 2. Deep learning (DL) is a subset of AI. Compared with the traditional machine learning technology, DL has obvious advantages. It can automatically extract more discriminative information features from data sets, and the extraction process is simpler, and the performance can be adjusted more easily and systematically 3.

Based on the analysis and discussion of relevant literature in recent years, this review will introduce the progress of DL in classification, biomarkers, prognosis prediction of lung cancer, and discuss the main problems faced by the application of deep learning in pathology, and predict its prospect of development.

## Overview of Deep learning

### Basic concepts and common models of Deep learning

AI is a way of using computers to simulate certain human thinking processes and intelligent behaviors (such as learning, reasoning, thinking, planning, etc.). DL is a subset of AI. It is a multi-layer neural network algorithm, which adjusts between layers and layers, automatically extracts multi-level low-level features, and then translates into more complicated abstract features to complete the learning of complex tasks 4. DL is one of the most critical factors for AI's success. It does not need too much manual intervention and has a strong degree of automation 5. Compared with traditional machine learning technology, DL has obvious advantages. DL can automatically extract richer and more discriminative information features from data sets, and the extraction process is simpler, and the performance can be adjusted more easily and systematically 3.

There are two representative models of DL model: (1) Unsupervised learning, i.e. deep belief networks (DBN) using unlabeled samples as training data sets. (2) Fully supervised learning, i.e. Convolutional Neural Network (CNN) using samples processed by artificial markers as training data sets. It is suitable for capturing local information in 2D or 3D images, and sliding window image classification based on image blocks is the basic application of CNN in medical image analysis. Full convolutional neural networks (FCN) is a special type of CNN, which is mainly used for image semantics segmentation. Now, CNN is moving towards a deeper and more accurate direction in order to better feature representation.

Migrating on the above-mentioned pre-trained network model is transfer learning, and only a few data sets are needed to complete the new classification task 6. The first strategy includes using pre-trained networks as feature extractors. The second strategy is to finetune pre-trained networks 7.

### Application Status of DL in Medical Field

In the medical field, DL can transform qualitative subjective image information into quantitative objective image information, and help clinicians to supplement clinical decision-making. CT, MRI and pathological images in medical images belong to structured data, and DL is easy to extract and learn efficiently. Therefore, DL has great potential in medical image analysis. In recent years, the deep neural network has shown the same accuracy as clinicians in the diagnosis of cutaneous malignant melanoma 8. In the field of ophthalmology, the depth neural network assessment of retinal fundus images shows high sensitivity and specificity, which is conducive to the detection of diabetic retinopathy 9. The development trend of DL is gradually applied to new fields, including lung cancer pathology.

In the past, the pathological detection and reporting of lung cancer was mainly undertaken by clinicopathologists. There were many mechanical repetitive tasks, so reading slices was time-consuming and inefficient, and there were geographical constraints. It is difficult to quantify image information because of its strong subjectivity. Therefore, the level of diagnosis is different and the rate of misdiagnosis is higher. Current studies show that DL-based lung cancer pathology overcomes these shortcomings by qualitative or quantitative analysis of tumor information such as cell morphology, histological texture features and distribution features of lung cancer pathological slices and immunohistochemical staining intensity of specific molecules. It has great potential for application and is expected to change the clinical workflow of pathological diagnosis of lung cancer in the future.

## Computer aided diagnosis of lung cancer pathology based on deep learning

### Application of CAD deep learning model in pathological classification of lung cancer

The lung cancer are most common mainly divided into two types according to pathology: non-small cell lung cancer (NSCLC) and small cell lung cancer (SCLC). NSCLC can be divided into three main histological types: squamous cell carcinoma, adenocarcinoma and large cell carcinoma. Because the treatment methods of the two kinds of lung cancer are very different, it is very important to classify the pathological types of lung cancer accurately in clinical work. DL can help pathologists detect lung cancer subtypes.

Exfoliative cytology is helpful for the early diagnosis of lung cancer, and most of them can also distinguish the pathological types of lung cancer. Teramoto et al. 10 analyzed the Pap staining images of 76 cases of lung cancer, including adenocarcinoma, squamous cell carcinoma and small cell carcinoma. Two kinds of CNNs are trained by using the original data and the enhanced data of the original data, and then the original image is input. The trained CNN model is used to extract features, and finally the cancer type is judged to evaluate the performance of the two kinds of CNNs. The results showed that the classification using enhanced data was more accurate, with a total accuracy rate of 71.1%, which was comparable with that of cytology technicians and pathologists.

Khosravi et al. 6 uses TensorFlow software running in the Mac system to compare different algorithms for differentiating squamous cell carcinoma and adenocarcinoma from two public databases of lung cancer: Stanford Tissue Microarray Database (TMAD) and The Cancer Genome Atlas (TCGA). A large number of images are trained in the database, and a small number of images are used to verify and test the model. Among them, Inception-V1 and Inception-V3, a new architecture of fine-tuned pre-trained CNN, can distinguish squamous cell carcinoma from adenocarcinoma in 100% high resolution magnified images of local lesions. The classification accuracy of full slice image fluctuates between 75% and 90%. Therefore, the strategy of fine-tuning the pre-training network may solve the serious lack of public medical data. Pathologists outline the local atypical lesions of lung tumors, which can significantly improve the accuracy of CNN classification. This human-computer cooperation model is more promising for early clinical application. Among them, Inception-V1 and Inception-V3, a new architecture of fine-tuned pre-trained CNN, can distinguish squamous cell carcinoma from adenocarcinoma in 100% high resolution magnified images of local lesions. The classification accuracy of full slice image fluctuates between 75% and 90%. Therefore, the strategy of fine-tuning the pre-training network may solve the serious lack of public medical data. Pathologists outline the local atypical lesions of lung tumors, which can significantly improve the accuracy of CNN classification. This human-computer cooperation model is more promising for early clinical application.

Hou et al. 11 combined the sliding window classification framework based on image blocks with the traditional DL method, improved 13 decision fusion models, trained a large number of image data from histopathological H&E staining images of TCGA, input test images, automatic analysis of the model, classified according to the morphological characteristics of images, output three results of lung squamous cell carcinoma, lung adenocarcinoma or mixed lung adenocarcinoma. By testing different algorithms, it shows that the classification accuracy of the improved model is higher than that of the pure CNN, which is close to that of pathologists. The improved model EM-Finetune-CNN-SVM has obvious advantages in dealing with complex mixed lung adenocarcinoma. Therefore, high performance algorithm is the key factor of CAD.

Coudray et al. 12 trained inception-v3 with a large number of image data in TCGA histopathological H&E staining image by manual outline of tumor area by pathologists. In this model, transfer learning is used, and a small number of images are used to verify and test the model. After Input test image and automatic analysis of the model, three results of normal tissue, lung squamous cell carcinoma or lung adenocarcinoma were output according to morphological characteristics and immunohistochemical staining intensity of the image. AUC reached 0.97, similar to pathologists. They also use image data outside TCGA to validate the model. AUC achieves a high level, which proves the universality and robustness of the model. They designed and validated that the feature extracted by DL from the tumor area manually outlined by pathologists can automatically label the tumor area by computer, which is comparable to the ability of pathologists to label the tumor area manually. Therefore, the automation ability of DL can reach a high level after proper training, which will significantly improve the efficiency of tumor recognition.

Daniel Bug et al. 13 present a deep learning workflow for the classification of hematoxylin and eosin stained histological whole-slide images of non-small-cell lung cancer. Extracted meta-features of the different tissue classes and then semi-automatic analysis was carried out. They got a state-of-the-art result with an average F1-score of 83%. This DL workflow supports large-scale analysis of tissue obtained in preclinical animal experiments, enables reproducible quantification of tissue classes and immune system markers. Xi Wang et al. 14 propose a Weakly Supervised Deep Learning for fast and effective classification on the whole slide lung cancer images. The classification accuracy of 939 WSI is up to 97.3%, The AUC of the public lung cancer WSIs dataset classification from The Cancer Genome Atlas (TCGA) is 85.6%. Among them, the causes of misclassification are mainly due to the poor differentiation of tumors and the low proportion of tumor tissue as a whole.

### “Performance of Deep Learning Model” VS Radiologist in lung cancer early detection and differential diagnosis

Computer-aided pathology and imaging diagnosis have become the focus of current research due to their high efficiency, time-saving and labor-saving. They also face problems such as model performance to be improved, data set to be expanded and standardized. Compared with the former, the latter has been studied earlier and more, and computer-aided CT nodal classification has been studied for more than 30 years 15, and the classification performance has increased year by year. In recent years, the classification accuracy of some models is even more than 90% 16. However, it is difficult to compare the deep learning model based on pathology with the deep learning model based on imaging for the early recognition of lung cancer and the classification performance of lung cancer directly, because up to now, there is no unified standard in the research of scholars, and the selected data set, validation set, intervention measures, adjustment measures are very different. Histopathology image analysis serves as the gold standard for cancer diagnosis, before diagnosis, CT image classification is the key step. It can be seen that they provide different information on the early diagnosis and subtype classification of lung cancer. In the future, if we can integrate the CT images, location information, pathological images, clinical features and other information of pulmonary nodules, and give full play to the advantages of imagers, pathologists, clinicians and computer scientists, so that people can have a deep understanding of lung cancer, the diagnosis of lung cancer will be more accurate, and the advantages of computer-aided diagnosis of lung cancer can really play out.

### Deep learning and prediction of lung cancer mutation genes

In clinical practice, tumors of the same pathological subtype may differ considerably at the molecular level and therefore respond differently to treatment. Different mutation states of driving genes have different TKI targeted therapies. The mutation status of epidermal growth factor receptor (EGFR) is critical for the selection of patients treated with erlotinib and gefitinib. 17. Coudray et al. 12 trained inception-v3 with a large number of image data from TCGA histopathological images. According to the results of gene sequencing data, the model was input to extract morphological features related to mutation. A small number of images were used to validate and test the model, and then the test image was input. After automatic analysis of the model, six frequently mutated genes - STK11, EGFR, FAT1, SETBP1, KRAS and TP53 of lung adenocarcinoma were successfully predicted according to the morphological characteristics of the image. The predicted AUC of EGFR mutation reached 0.83, and then the universality of the model was verified. AUC could reach 0.75. The appearance of this model can preliminarily predict gene mutation by only inputting H&E stained images, overcome the shortcomings of human naked eye recognition and summary of features, and has the advantages of low cost and high efficiency.

### Application of deep learning in prognosis of lung cancer

CAD prognosis is a promising machine learning algorithm. It is an important supplement to the general guideline of TNM staging of lung cancer, with the advantages of simplicity and rapidity. The parameters obtained can provide prognostic information. It is of great significance for clinical diagnosis and treatment of lung cancer, and also provides a new perspective for understanding the biological mechanism of cancer.

DL can be linked with traditional machine learning to obtain quantitative information useful for the prognosis of lung cancer patients. Li et al. 18 Using trained CNN, pathological images of 205 NSCLC patients were transformed into maps of the distribution of lymphocytes, tumor cells and stromal cells. Then, Bayesian hidden Potts model and prior Markov random field (MRF) machine learning model are improved to Bayesian hidden Potts mixture model, which can effectively fuse and observe multiple feature information. When the model is input into the cell distribution map, the parameters representing the intensity of cell-to-cell interaction can be output, and the interaction among tumor cells, stromal cells and lymphocytes can be quantified. Cox regression model analysis shows that the intensity of interaction between tumor cells and stromal cells is significantly correlated with the patient's good prognosis (*p* = 0.005).

DL can establish links with biological network analysis system and hopefully obtain quantitative information which is helpful for clinical decision-making. Choi et al. 19 trained 510 clinicopathological data of lung adenocarcinoma in Gene Expression Omnibus and validated the gene co-expression network. According to the output gene spectrum, 20 representative genes which were most closely related to patient survival were selected and input into DL. According to the expression patterns of various genes extracted, NetScore i.e. Risk Stratification Model was exported, which is more predictive than the traditional Cox proportional hazard model, may help identify patients requiring neoadjuvant chemotherapy.

Mezheyeuski et al. 20 used multiple imaging systems based on deep learning to quantify and locate important subtypes of immune cells in 57 NSCLC patients, and quantify the infiltration pattern of immune cells associated with prognosis. The results showed that the more abundant lymphocyte types, the higher the ratio of cytotoxic T cells in the tumor nest to their matrix, the larger the ratio of regulatory T cells to effector T cells in the same region, and the less CD8 positive regulatory T cells aggregated near the tumor, the better the prognosis of patients. However, due to its small amount of data and lack of representativeness, this conclusion needs further verification.

Microvessel density (MVD) is usually used as an alternative indicator of angiogenesis. MVD is an important prognostic factor for non-small cell lung cancer 21. Yi et al. 22 used the microvessel-related features labeled manually by pathologists in histopathological H&E staining images as the data training set of FCN. After fine-tuning, the trained FCN produced the final FCN model, which quantifies the microvessel characteristics. By inputting the pathological H&E staining images, the total microvessel area and the percentage of tumor cells around the microvessel could be output. The automation of this model can be markedly improved by using microvasculature-stained antibodies to delineate microvasculature.

Shidan Wang et al. 23 developed an automated cell type classification pipeline, named ConvPath, classifying tumor cell, stromal cell, and lymphocyte of lung adenocarcinoma with high accuracy up to 92.9% and 90.1% in training and independent testing datasets, respectively. The ConvPath outputs the microenvironment characteristics of tumor according to the spatial distribution characteristics of different kinds of cells, which are proved to be independent prognostic factors of lung adenocarcinoma. A DL-based analysis method could predict patient survival by analyzing 48 cell spatial organization of lung adenocarcinoma H&E staining images. The late survival rate of high-risk group was significantly lower than that of low-risk group, adjusted risk ratio is 2.23 [95% CI 1.37-3.65]. What's more, it related to gene expression of biological pathways, including T-cell receptor (TCR), programmed cell death protein 1 (PD1) and extracellular matrix organization pathway 24. Elastic net-Cox proportional hazards models was used to analyze the HE staining features of stage I adenocarcinoma and squamous cell carcinoma of the lung, which were related to the survival outcome in both TCGA and TMAD 25.

### Application prospects of deep learning model

The selection and processing of data sets is the promotion space of CAD. Yi et al. 22 showed that by increasing the number of image data trained by FCN, the annotation accuracy can be enhanced and the quality of data set can be improved. Therefore, we look forward to a more authoritative public pathological image database with a large number of data sets in order to extract more abstract features and cover more comprehensive features. For most lung cancers, the phase equilibrium state of various data sets can improve accuracy and reduce misjudgement. In practice, data increase, pre-training, fine-tuning and introducing attention mechanism are usually used to solve these problems.

The problems of image itself affect the results of CAD. Coudray et al. 12 analyzed the causes of CNN misclassification: 1) low degree of undifferentiated tumors themselves; 2) unknown features of the model, such as blood clots, blood vessels, inflammation, necrosis, lung collapse, fibrosis scar and bronchial cartilage, interfered the classification. The different responses of DL in practice may be due to genetic heterogeneity. Further research is needed to clarify the efficiency of DL in detecting heterogeneous images 6.

High performance algorithms are closely related to the accuracy of CAD. Khosravi et al. 6 proposed that the factors affecting the accuracy of CNN classification depend not only on the size of the data set, but also on the complexity, algorithm architecture and noise. Therefore, the optimized algorithm has been tested by repeated data. If we can achieve the same level as clinicopathologist, the CNN with high robustness will be put into clinical use just around the corner.

At present, there is no research on the pathological grading of lung cancer in the field of pathological CAD. The pathological grading of tumors according to their pathological morphology can reflect the malignant degree of tumors and provide basis for clinical treatment and prognosis 26. For example, the recognition of mitotic cells in tumors is of great significance in the grading of tumors.

Improving the accuracy and robustness of DL in lung cancer pathological CAD is the joint efforts of computer scientists and pathologists. We look forward to more accurate DL models and methods.

### Advantages and shortcomings of Deep Learning Model

The research on DL model can show its advantages and shortcomings in Table 1. As for the advantages, the computer-aided diagnosis of lung cancer pathology based on DL is time-saving, manpower saving, high accuracy, comprehensive analysis, which is conducive to precise treatment and strong ability to predict the prognosis of patients, especially the patients with lung adenocarcinoma 18-20, 22, 23-25, and can be used to evaluate the curative effect 13, 23. In terms of shortcomings, lung cancer pathological images have strong heterogeneity 6, 14, the data set of model training is small 11, 20, the histological features covered by the data set are not comprehensive 12, 22-24, the model is difficult to comprehensively consider all pathological characteristics 10, and the training and verification of public data sets are difficult to be extended to clinical practice 19, 25.

### Deep Learning model for Predicting Lung Cancer Treatment Response

Daniel Bug et al. 13 pave the way towards discovery of novel features predicting response in translational immune-oncology research. Tumors with a high fraction of necrotic tissue and a low fraction of tumor are likely to have responded to the treatment, as the necrosis can be explained as decay of tumor tissue. In other studies, it is suggested that the spatial distribution of pathological images may indicate the efficacy of immunotherapy 23.

## Application of DL in pathological research of lung cancer

With the increasing awareness of the relationship between gene mutation and immune factors and cancer development, more and more studies have been carried out to identify biomarkers of targeted molecular therapy and immunotherapy. Tumor pathology has a broad prospect of scientific research. The researches of DL in biomarkers of lung cancer is of great significance to explore the characteristics, pathological process and search for new therapeutic targets, as well as to the individualized treatment of patients.

Artificial intelligence improves the efficiency of molecular research. Hamilton 27 et al. developed and validated a computer-aided image analysis method called Tissue Mark for the identification, annotation and analysis of H&E stained tissue slices of tumors. This method focuses on the application of NSCLC. It uses high-efficiency image processing, quantitative feature analysis and pattern recognition technology to recognize the tumor area in slices, calculate the percentage of tumors, and extract and detect nucleic acids easily in high-tumor areas. This model can significantly accelerate the time of manual selection of tumor regions in large cohort studies or clinical trials, and help to identify molecular biomarkers 27.

DL promotes the study of immune factors in lung cancer. Mezheyeuski et al. 20 trained and validated a deep learning model by transforming the immunofluorescence images of 57 NSCLC patients with multiple immunofluorescent staining images. The test images were input into the model then analyzed automatically, and three results were output: immune cells (cells expressing immune markers), tumor cells (cells expressing high CK) or negative cells (non-tumor cells not expressing immune markers). output three results of immune cells (cells expressing immune markers), tumor cells (cells expressing high CK) or labeled negative cells (non-tumor cells not expressing immune markers). Semi-quantitative analysis of five immune markers (CD8, CD20, CD4, FOXP3 and CD45RO) and lymphocytes was carried out. The results show that the digital semi-quantitative method based on DL system is equivalent to or better than the traditional visual semi-quantitative method.

## Conclusions

To summarize, DL-based lung cancer pathology CAD and scientific research has shown good performance and great potential. But at present, DL is still in its infancy, there are many problems to be solved, the accuracy of processing is still to be improved, and some complex functions cannot be achieved. Both clinical work and scientific research work are very rigorous work, need higher requirements. In addition, the authoritative lung cancer pathology database is scarce, which makes it difficult to explain the universality of the training model. DL cannot be directly applied to clinical research practice and clinical work right now. We expect that the DL-based lung cancer pathology CAD and scientific research will be continuously improved with the deepening of the research, and hopefully more in-depth participation in all aspects of lung cancer pathology, to help doctors and researchers analyze and process these massive data.

## Figures and Tables

**Table 1:** Advantages and shortcomings of Deep Learning Model

Topic	Lung Cancer Subtype	Pathological images	Method	Year	Advantages	Shortcomings	Ref.
**Lung cancer classification**	ADC SCC SCLC	CytologicalPap staining images	CNN trained by original data vs. enhanced data	2017	Trained by enhanced data improve classification accuracy	Limited in comprehensively classifying cells and arrays of cells	10
	ADC SCC	H&E and IHC images	CNN pre-trained by Inception-V1 and Inception-V3, with pathologists outlining the local atypical lesions	2018	Human-computer cooperation model improve the classification accuracy, reduce the false rate, and accelerate training speed by limited public medical data	Limited in detection of heterogeneity through digital images	6
	SCC ADC MixedADC	H&E and IHC images	improved model: EM-Finetune-CNN-SVM	2016	Obvious advantages in dealing with complex mixed ADC	Limited in small scale pathology data	11
	Normal tissue SCC ADC	Frozen tissues, formalin-fixed paraffin-embedded tissues and biopsies	CNN pre-trained by Inception-V3, with pathologists outlining tumor area	2018	Speed up diagnosis and classification during intraoperative consultation	Limited in detection of diversity and heterogeneity of tissues, need trained by a wider range of histologic features	12
	Normal tissue SCC ADC SCLC	Whole slide image (WSI)	weakly supervised learning FCN	2019	Minimize annotation cost More efficient and precise diagnosis	Not properly deal with ambiguous regions in WSIs due to complex technique variations (e.g., variations of color/texture) and biological heterogeneities	14
	NSCLC	H&E images	parameter-efficient network structure	2019	Quantification of tissue classes and immune system markers	-	13
**prediction of lung cancer mutation genes**	ADC	frozen tissues, formalin-fixed paraffin-embedded tissues and biopsies	CNN pre-trained by Inception-V3, with pathologists outlining tumor area	2018	Provide accurate diagnosis, which can be beneficial to the treatment of patients	-	12
**prognosis**	-	H&E images	Bayesian hidden Potts mixture model	2019	1. Low cost 2. Quantify the interaction energy between different types of cells 3. Distinguish clinically meaningful patterns from the background area via a Markov random field model	-	18
	ADC	Clinicopathological features	Risk Stratification Model	2018	1. More predictive in early stage subgroups (stage IA/IB) 2. Beneficial to identifying patients requiring neoadjuvant chemotherapy	Lack of clinical application evidence	19
	NSCLC	IHC images	fluorescence-based multiplexed immunohistochemical method in combination with a multispectral imaging system	2018	Guide clinical decisions in immunotherapy	Small amount of data and lack of representativeness	20
	ADC	H&E images	FCN after fine-tuning	2018	automated microvessel detection in H&E stained pathology images	A false positive problem for background regions where a large number of blood cells appear. 1. Comprehensive cell types 2. Tumor microenvironment is related to gene expression of biological pathways	22
	ADC	H&E images	Histology-based Digital (HD)-Staining, a DL-based model	2020	Comprehensive cell types Tumor microenvironment is related to gene expression of biological pathways	1. Morphological and intensity features of nuclei was not included. 2. Some special Structures was not included.	24
	stage I ADC SCC	H&E images	Elastic net-Cox proportional hazards models	2016	Image features are more comprehensive	Pathological features can not represent pathological sections in actual work	25
	ADC	frozen and Formalin-Fixed, Paraffin-Embedded (FFPE) slides	ConvPath	2019	1. Save time 2. It may indicate the effect of immunotherapy	1. The cell type is not complete, so it is not allowed to type a cell specifically 2. The spatial distribution of cells is not comprehensive	23

## Abbreviations

CAD: computer-aided diagnosis
AI: artificial intelligence
DL: Deep learning
DBN: Deep learning
CNN: Convolutional Neural Network
FCN: Full convolutional neural networks
CT: computer tomography
MRI: magnetic resonance imaging
NSCLC: non-small cell lung cancer
SCLC: small cell lung cancer
TMAD: Tissue Microarray Database
TCGA: The Cancer Genome Atlas
AUC: area under curve
MRF: markov random field
MVD: microvessel density
